# Supplementary material for: Heat adaptation of phage T7 under an extended genetic code
Source: Virus Evol. 2021 Dec 1;7(2):veab100. doi: 10.1093/ve/veab100 (PMC8923235; doi:10.1093/ve/veab100)
Supplement: veab100_Supp [file veab100_supp.zip › Sequencing_Analysis.zip!/Sequencing_Analysis/S2/evidence/SNP_9.html]

BRESEQ :: Evidence


| Predicted mutation | | | | | | |
| --- | --- | --- | --- | --- | --- | --- |
| evidence | seq id | position | mutation | annotation | gene | description |
| RA | NC\_001604.1 | 28,691 | A→C | intergenic (–/–) | – / – | –/– |

| Read alignment evidence... | | | | | | | | | | | |
| --- | --- | --- | --- | --- | --- | --- | --- | --- | --- | --- | --- |
|  | seq id | position | | ref | new | freq | score (cons/poly) | reads | annotation | genes | product |
| \* | NC\_001604.1 | 28,691 | 0 | A | C | 99.5% | 14781.0 / NA | 4760 | intergenic (–/–) | *–/–* | –/– |
| Reads supporting (aligned to +/- strand):  **ref** base A (13/12);  **new** base C (2347/2376);  **total** (2367/2393) | | | | | | | | | | | |
| Fisher's exact test for biased strand distribution *p*-value = 8.44e-01 | | | | | | | | | | | |
| Kolmogorov-Smirnov test that lower quality scores support variant *p*-value = 9.99e-01 | | | | | | | | | | | |

Only 100 of 4838 total aligned reads displayed.  

`AGGTATGGGCTATCGTGCAGCAACCACTCAGGCCGAACAGCCAAGGTCAAGCCTATTGGACACCATTGGTCGGTTCGCTAAGGCTGGTGCCGATATGTATACCGCTAAGGAACAACGAGCACGAGACCTAGCTGATGAACGCTCTAACGAGATTATCCGTAAGCTGACCCCTGAGCAACGTCGAGAAGCTCTCAACAACGGGACCCTTCTGTATCAGGATGACCCATA‑‑CGCTATGGAAGCACTCCGAGTCAAGACTGGTCGTAACGCTGCGTATCTTGTGGACGATGACGTTATGCAGAAGATAAAAGAGGGTGTCTTCCGTACTCGCGAAGAGATGGAAGAGTATCGCCATAGTCGCCTTCAAGAGGGCGCTAAGGTATACGCTGAGCAGTTCGGCATCGACCCTGAGGACGTTGATTATCAGCGTGGTTTCAACGGGGACATTACCGAGCGTAACATCTCGCTGTATGGTGCGCATGATAACTTCTTGAGCCAGCAAGCTCAGAAGGGCGCTATCATGAACAGCCGAGTGGAACTCAACGGTGTCCTTCAAGACCCTGATATGCTGCGTCGTCCAGACTCTGCTGACTTCTTTGAGA  >  NC_001604.1/28393‑28991`  

                                                                                                                                                                                                                                                                                                            `|`                                                                                                                                                                                                                                                                                                              
`aGGTATGGGCTATCGTGCAGCAACCACTCAGGCCGAACAGCCAAGGTCAAGCCTATTGGACACCATTGGTCGGTTCGCTAAGGCTGGTGCCGATATGTATACCGCTAAGGAACAACGAGCACGAGACCTAGCTGATGAACGCTCTAACGAGATTATCCGTAAGCTGACCCCTGAGCAACGTCGAGAAGCACTCAACAACGGGACCCTTCTGTATCAGGATGACCCATA‑‑CGCTATGGAAGCACTCCGAGTCAAGACTGGTCGTAACGCTGCGTATCTTGTGGACGATGACGTTATGcagcag                                                                                                                                                                                                                                                                                                            >  1:1961/1‑301 (MQ=255)`  
       `ggCTATCGTGCAGCAACCACTCAGGCCGAACAGCCAAGGTCAAGCCTATTGGACACCATTGGTCGGTTCGCTAAGGCTGGTGCCGATATGTATACCGCTAAGGAACAACGAGCACGAGACCTAGCTGATGAACGCTCTAACGAGATTATCCGTAAGCTGACCCCTGAGCAACGTCGAGAAGCTCTCAACAACGGGACCCTTCTGTATCAGGATGACCCATA‑‑CGCTATGGAAGCACTCCGAGTCAAGACTGGTCGTAACGCTGCGTATCTTGTGGACGATGACGTTATGCAGCAGATAAAag                                                                                                                                                                                                                                                                                                     <  1:693907/301‑1 (MQ=255)`  
       `acagaTCGTGCAGCAACCACTCAGGCCGAACAGCCAAGGTCAAGCCTATTGGACACCATTGGTCGGTTCGCTAAGGCTGGTGCCGATATGTATACCGCTAAGGAACAACGAGCACGAGACCTAGCTGATGAACGCTCTAACGAGATTATCCGTAAGCTGACCCCTGAGCAACGTCGAGAAGCTCTCAACAACGGGACCCTTCTGTATCAGGATGACCCATA‑‑CGCTATGGAAGCACTCCGAGTTAAGACTGGTCGTAACGCTGCGTATCTTGTGGACGATGACGTTATGCAGCAGATAAAag                                                                                                                                                                                                                                                                                                     <  1:211498/297‑1 (MQ=255)`  
         `agaTCGTGCAGCAACCACTCAGGCCGAACAGCCAAGGTCAAGCCTATTGGAAACCATTGGTCGGTTCGCTAAGGCTGGTTCCGATATGTATACCGCTAAGGAACAACGAGCACGAGACCTAGCTGATGAACGCTCTAACGAGATTATCCGTAATCTGACCCCTGAGCAACGTCGAGAAGCTCTCAACAACGGGACCCTTCTGTATCAGGATGACCCATA‑‑CGCTATGGAAGCACTCCGAGTCAAGACTGGTCGTAACGCTGCGTATCTTGTGGACGATGACGTTATGCAGCAGATAAAagag                                                                                                                                                                                                                                                                                                   <  1:320300/299‑1 (MQ=255)`  
           `aTCGTGCAGCAACCACTCAGGCCGAACAGCCAAGGTCAAGCCTATTGGACACCATTGGTCGGTTCGCTAAGGCTGGTGCCGATATGTATACCGCTAAGGAACAACGAGCACGAGACCTAGCTGATGAACGCTCTAACGAGATTATCCGTAAGCTGACCCCTGAGCAACGTCGAGAAGCTCTCAACAACGGGACCCTTCTGTATCAGGATGACCCATA‑‑CTCTATGGAAGCACTCCGAGTCAAGACTGGTCGTAACGCTGCGTATCTTGTGGACGATGACGTTATGCAGCAGATAAAagctgt                                                                                                                                                                                                                                                                                                 >  1:685607/1‑297 (MQ=255)`  
           `aTCGTGCAGCAACCACTCAGGCCGAACAGCCAAGGTCAAGCCTATTGGACACCATTGGTCGGTTCGCTAAGGCTGGTGCCGATATGTATACCGCTAAGGAACAACGAGCACGAGACCTAGCTGATGAACGCTCTAACGAGATTATCCGTAAGCTGACCCCTGAGCAACGTCGAGAAGCTCTCAACAACGGGACCCTTCTGTATCAGGATGACCCATA‑‑CGCTATGGAAGCACTCCGAGTCAAGACTGGTCGTAACGCTGCGTATCTTGTGGACGATGACGTTATGCAGCAGATAAAAGAggg                                                                                                                                                                                                                                                                                                 >  1:605408/1‑301 (MQ=255)`  
             `ataagagacagcACTCAGGCCGAACAGCCAAGGTCAAGCCTATTGGACACCATTGGTCGGTTCGCTAAGGCTGGTGCCGATATGTATACCGCTAAGGAACAACGAGCACGAGACCTAGCTGATGAACGCTCTAACGAGATTATCCGTAAGCTGACCCCTGAGCAACGTCGAGAAGCTCTCAACAACGGGACCCTTCTGTATCAGGATGACCCATA‑‑CGCTATGGAAGCACTCCGAGTCAAGACTGGTCGTAACGCTGCGTATCTTGTGGACGATGACGTTATGCAGCAGATAAAAGAGGgtg                                                                                                                                                                                                                                                                                               <  1:620720/290‑1 (MQ=255)`  
                   `gcaACCACTTAGGCCGAACAGCCAAGGTCAAGCCTATTGGACACCATTGGTCGGTTCGCTAAGGCTGGTGCCGATATGTATACCGCTAAGGAACAACGAGCACGAGACCTAGCTGATGAACGCTCTAACGAGATTATCCGTAAGCTGACCCCTGAGCAACGTCGAGAAGCTCTCAACAACGGGACCCTTCTGTATCAGGATGACCCATA‑‑CGCTATGGAAGCACTCCGAGTCAAGACTGGTCGTAACGCTGCGTATCTTGTGGACGATGACGTTATGCAGCAGATAAAagctgtctcttata                                                                                                                                                                                                                                                                                         >  1:781301/1‑289 (MQ=255)`  
                    `ctcggagatttgtataagagacagggTCAAGCCTATTGGACACCATTGGTCGGTTCGCTAAGGCTGGTGCCGATATGTATACCGCTAAGTAACAACGAGCACGAGACCTAGCTGATGAACGCTCTAACGAGATTATCCGTAAGCTGACCCCCGAGCAACGTCGAGAAGCTCTCAACAACGGGACCCTTCTGTATCAGGATGACCCATA‑‑CGCTATGGAAGCACTCCGAGTCAAGACTGGTCGTAACGCTGCGTATCTTGTGGACGATGACGTTATGCAGCAGATAAAAGAGGGTGTCTTCCg                                                                                                                                                                                                                                                                                        <  1:657199/277‑1 (MQ=255)`  
                       `ccAATCAGGCCGAACAGCCAAGGTCAAGCCTATTGGACACCATTGGTCGGTTCGCTAAGGCTGGTGCCGATATGTATACCGCTAAGGAACAACGAGCACGAGACCTAGCTGATGAACGCTCTAACGAGATTATCCGTAAGCTGACCCCTGAGCAACGTCGAGAAGCTCTCAACAACGGGACCCTTCTGTATCAGGATGACCCATA‑‑CGCTATGGAAGCACTCCGAGTCAAGACTGGTCGTAACGCTGCGTATCTTGTGGACGATGACGTTATGCAGCAGATAAAAGAGGGTGTCTTCCGTAc                                                                                                                                                                                                                                                                                     <  1:355894/301‑1 (MQ=255)`  
                                 `acagcAGCCAAGGTCAAGCCTATTGGACACCATTGGTCGGTTCGCTAAGGCTGGTGTCGATATGTATACCGCTAAGGAACAACGAGCACGAGACCTAGCTGATGAACGCTCTAACGAGATTATCCGTAAGCTGACCCCTGAGCAACGTCGAGAAGCTCTCAACAACGGGACCCTTCTGTATCAGGATGACCCATA‑‑CGCTATGGAAGCACTCCGAGTCAAGACTGGTCGTAACGCTGCGTATCTTGTGGACGATGACGTTATGCAGCAGATAAAAGAGGGTGTCTTCCGTACTCGCGAagag                                                                                                                                                                                                                                                                           <  1:766991/297‑1 (MQ=255)`  
                                        `aagagacagaGCCTATTGGACACCATTGGTCGGTTCGCTAAGGCTGGTGCCGATATGTATACCGCTAAGGAACAACGAGCACGAGACCTAGCTGATGAACGCTCTAACGAGATTATCCGTAAGCTGACCCCTGAGCAACGTCGAGAAGCTCTCAACAACGGGACCCTTCTGTATCAGGATGACCCATA‑‑CGCTATGGAAGCACTCAGAGTCAAGACTGGTCGTAACGCTGCGTATCTTGTGGACGATGACGTTATGCAGCAGATAAAAGAGGGTGTCTTCCGTACTCGCGAAGAGATGGAag                                                                                                                                                                                                                                                                    <  1:139971/292‑1 (MQ=255)`  
                                          `agggTCAAGCCTATTGGACACCATTGGTCGGTTCGCTAAGGCTGGTGCCGATATGTATACCGCTAAGGAACAACGAGCACGAGACCTAGCTGATGAACGCTCTAACGAGATTATCCGTAAGCTGACCCCTGAGCAACGTCGAGAAGCTCTCAACAACGGGACCCTTCTGTATCAGCATGACCCATACGCGCTATGGAAGCACTCCGAGTCAAGACTGGTCGTAACGCTGCGTATCTTGTGGACGATGACGTTATGCAGCAGATAAAATAGGGTGTCTTCCGTACTCGCGAAGAGATGGAag                                                                                                                                                                                                                                                                    <  1:371621/299‑1 (MQ=255)`  
                                            `ggTCAAGCCTATTGGACACCATTGGTCGGTTCGCTAAGGCTGGTGCCGATATGTATACCGCTAAGGAACAACGAGCACGAGACCTAGCTGATGAACGCTCTAACGAGATTATCCGTAAGCTGACCCCTGAGCAACGTCGAGCAGCTCTCAACAACGGGACCCTTCTGTATCAGGATGACCCATA‑‑CTCTATGGAAGCACACCGAGTCAAGACTGGGCGTAACGCTGCTTATCTTGTGCACGATGGCGTTATGCAGCCGATAAAAGAGGGTTGCTTCCGTACTCGCGCAGAGATGGCAGATTa                                                                                                                                                                                                                                                                >  1:285983/1‑301 (MQ=255)`  
                                            `ggTCAAGCCTATTGGACACCATTGGTCGGTTCGCTAAGGCTGGTGCCGATATGTATACCGCTAAGGAACAACGAGCACGAGACCTAGCTGATGAACGCTCTAACGAGATTATCCGTAAGCTGACCCCTGAGCAACGTCGAGAAGCTCTCAACAACTGGACCCTTCTGTATCAGGATGACCCATA‑‑CGCCATGGAAGCACTCCGAGTCAAGACTGGTCGTAACGCTGCGTATCTTGTGGACGATGACGTTATGCAGCAGATAAAAGAGGGTGTCTTCCGTACTCGCGAAGAGATGGAAGAGTa                                                                                                                                                                                                                                                                >  1:645277/1‑301 (MQ=255)`  
                                             `gtaaaGCCTATTGAACACCATTGCTGGGGTTGTCAAGGCTGGTGACGATAAGTACACGCCTAAGGAACAACGAGCACGAGTCCTAGCTGATGAACCCTCTAACGAGATTATCCGTAAGCTGACCCCTGAGTAACGTCGAGAAGCTCTCAACAACTGGACCCTTCTGTATCAGGATGACCCATA‑‑CGCTATGGAAGCACTCCGAGTCAAGACAGGTCGTAACGCTGCGTATCTTGTGGACGATGACGTTATGCAGCAGATAAAAGAGGGTGTCTTCCGTACTCGCGAAGAGATGGAAGAGTAt                                                                                                                                                                                                                                                               <  1:562574/298‑1 (MQ=255)`  
                                                    `cTATTGGACACCATTGGTCGGTTCGCTAAGGCTGGTGCCGATATGTATACCGCTAAGGAACAACGAGCACGAGACCTAGCTGATGAACGCTCTAACGAGATTATCCGTAAGCTGACCCCTGAGCAACGTCGAGAAGCTCTCAACAACGGGACCCTTCTGTATCAGGATGACCCATA‑‑CGCTATGGAAGCACTCCGAGTCAAGACTGGTCGTAACTCTGCGTATCTTGTGGACGATGACGTTATGCAGCAGATAAAAGAGGGTGTCTTCCGTACTCGCGAAGAGATGGAAGAGTATCGCCATa                                                                                                                                                                                                                                                        >  1:305723/1‑301 (MQ=255)`  
                                                      `aTTGGACACCATTGGTCGGTTCGCTAAGGCTGGTGCCGATATGTATACCGCTAAGGAACAACGAGCACGAGACCTAGATGATGAACGCTCTAACGAGATTATCCGTAAGCTGACCCCTGAGCAACGTCGAGAAGCTCTCAACAACGGGACCCTTCTGTATCAGGATGACCCATA‑‑CGCTATGGAAGCACTCCGAGTCAAGACTGGTCGTAACGCTGCGTATCTTGTGGACGATGACGATATGCGGCAGATAAAAGAGGGTGTCTTCCGTACTCGCGAAGAGATGGAagctgtctcttataca                                                                                                                                                                                                                                                      >  1:503008/1‑287 (MQ=255)`  
                                                       `ttGGACACCATTGTTCGGTTCGCTAAGGCTGGTGCCGATATGTATACCGCTAAGGAACAACGAGCACGAGACCTAGCTGATGAACGCTCTAACGAGATTATCCGTAAGCTGATCCCTGAGCAACGTCGAGAAGCTCTCAACAACGGGACCCTTCTGTATCAGGATGACCCATA‑‑CGCTATGGAAGCACTCCGAGTCAAGACTGGTCGTAACGCTGCGTATCTTGTGGACGATGACGTTATGCAGCAGATAAAAGAGGGTGTCTTCCGTACTCGCGAAGAGATGGAAGAGTATCGCCATAGTc                                                                                                                                                                                                                                                     <  1:549381/301‑1 (MQ=255)`  
                                                                `gtgggctcggagatgtgtataagagacagtatGTATACCGCTAAGGAACAACGAGCACGAGACCTAGCTGATGAACGCTCTAACGAGATTATCCGTAAGCTGACCCCTGAGCAACGTCGAGAAGCTCTCAACAACGGTACCCTTCTGTATCAGGATGACCCATA‑‑CGCTATGGAAGCACTCCGAGTCAAGACTGGTCGTAACGCTGCGTATCTTGTGGACGATGACGTTATGCAGCAGATAAAAGAGGGTGTCTTCCGTACTCGCGAAGAGATGGAAGAGTATCGCCATAGTCGCCTTCAag                                                                                                                                                                                                                                            <  1:652049/272‑1 (MQ=255)`  
                                                                `acaggTCGGTTCGCTAAGGCTGGTGCCGATATGTATACCGCTAAGGAACAACGAGCACGAGACCTAGCTGATGAACGCTCTAACGAGATTATCCGTAAGCTGACCCCTGAGCAACGTCGAGAAGCTCTCAACAACGGGACCCTTCTGTATCAGGATGACCCATA‑‑CGCTATGGAAGCACTCCGAGTCAAGACTGGTCGTAACGCTGCGTATCTTGTGGACGATGACGTTATGCAGCAGATAAAAGAGGGTGTCTTCCGTACTCGCGAAGAGATGGAAGAGTATCGCCATAGTCGCCTTCAag                                                                                                                                                                                                                                            <  1:676149/298‑1 (MQ=255)`  
                                                                  `gggctcggagatgtgtataagagacagtatGTATACCGCTAAGGAACAACGAGCACGAGACCTAGCTGATGAACGCTCTAACGAGATTATCCGTAAGCTGACCCCTGAGCAACGTCGAGAAGCTCTCAACAACGGGACCCTTCTGTATCAGGATGACCCATA‑‑CGCTATGGAAGCACTCCGAGTCAAGACTGGTCGTAACGCTGCGTATCTTGTGGACGATGACGTTATGCAGCAGATAAAAGAGGGTGTCTTCCGTACTCGCGAAGAGATGGAAGAGTATCGCCATAGTCGCCTTCAagag                                                                                                                                                                                                                                          <  1:23372/274‑1 (MQ=255)`  
                                                                        `gTTCGCTACGGCTGGTGCCGATATGTATACCGCTAAGGAACAACGAGCACGAGACCTAGCTGATGAACGCTCTAACGAGATTATCCGTAAGCTGACCCCTGAGCAACGTCGAGAAGCTCTCAACAACGGGACCCTTCTGTATCAGGATGACCCATA‑‑CGCTATGGAAGCACTCCGAGTCAAGACTGGTCGTAACGCTGCGTATCTTGTGGACGATGACGTTATGCAGCAGATAAAAGAGGGTGTCTTCCGTACTCGCGAAGAGATGGAAGAGGATCGCCATAGTCGCCTTCAAGAGGgcgcg                                                                                                                                                                                                                                    >  1:154544/1‑300 (MQ=255)`  
                                                                           `cGCTAAGGCTGGTGCCGATATGTATACCGCTAAGGAACAACGAGCACGAGACCTAGCTGATGAACGCTCTAACGAGATTATCCGTAAGCTGACCCCTGAGCAACGTCGAGAAGCTCTCAACAACGGGACCCTTCTGTATCAGGATGACCCATA‑‑CGCTATGGAAGCACTCCGAGTCAAGACTGGTCGTAACGCTGCGTATCTTGTGGACGATGACGTTATGCAGCAGATAAAAGAGGGTGTCTTCCGTACTCGCGAAGAGATGGAAGAGTATCGCCATAGTCGCCTTCAAGAGGGCGCTAAg                                                                                                                                                                                                                                 >  1:166260/1‑301 (MQ=255)`  
                                                                            `gagatttgtataagagacaggTATACCGCTAAGGAACAACGAGCACGAGACCTAGCTGATGAACGCTCTAACGAGATTATCCGTAAGCTGACCCCTGAGCAACGTCGAGAAGCTCTCAACAACGGGACCCTTCTGTATCAGGATGACCCATA‑‑CGCTATGGAAGTACTCCGAGTCAAGACTGGTCGTAACGCTGCGTATCTTGTGGACGATGACGTTATGCAGCAGATAAAAGAGGGTGTCTTCCGTACTCGCGAAGAGATGGAAGAGTATCGCCATAGTCGCCTTCAAGAGGGCGCTAAgg                                                                                                                                                                                                                                <  1:326767/281‑1 (MQ=255)`  
                                                                              `taagagacaggCCGATATGTATACCGCTAAGGAACAACGAGCATGAGACCTAGCTGATGAACGCTCTAACGAGATTATCCGTAAGCTGACCCCTGAGCAACGTCGAGAAGCTCTCAACAACGGGACCCTTCTGTATCAGGATGACCCATA‑‑CGCTATGGAAGCACTCCGAGTCAAGACTGGTCGTAACGCTGCGTATCTTGTGGACGATGACGTTATGCAGCAGATAAAAGAGGGTGTCTTCCGTACTCGCGAAGAGATGGAAGAGTATCGCCATAGTCGCCTTCAAGAGGGCGCTAAGGta                                                                                                                                                                                                                              <  1:757744/291‑1 (MQ=255)`  
                                                                                  `gCTGGTGACGATATGTATACCGCTAAGGAACAACGAGCACGAGACCTAGCTGATGAACGCTCTAACGAGATTATCCGTAAGCTGACCCCTGAGCAACGTCGAGAAGCTCTCAACAACGGGACCCTTCTGTAT‑AGGATGACCCATA‑‑CGGTATGGAAGCACTCCGAGTCAAGACTGGTCGTAACGCTGCGTATCTTGTGGACGATGACGTTATGCAGCAGATAAAAGAGGGTGTCTTCCGTACTCGCGAAGAGATGGAAGAGTATCGCCATAGTCGCCTTCAAGAGGGCGCTAAGGTATACGc                                                                                                                                                                                                                         <  1:183155/301‑1 (MQ=255)`  
                                                                                   `gtGGTGCCGATATGTATACCGCTAAGGAACAACGAGCACGAGACCTAGCTGATGAACGCTCTAACGAGATTATACGTAAGCTGACCCCTGAGCAACGTCGAGAAGCTCTCAACAACGGGACCCTTCTGTATCAGGATGACCCATA‑‑CGCTATGGAAGCACTCCGAGTCAAGACTGGTCGTAACGCTGCGTATCTTGTGGACGATGACGTTATGCAGCAGATAAAAGAGGGTGTCTTCCGTACTCGCGAAGAGATGGAAGAGTATCGCCATAGTCGCCTTCAAGAGGGCGCTAAGGTATACGc                                                                                                                                                                                                                         <  1:378507/300‑1 (MQ=255)`  
                                                                                   `cTGGTGCCGATATGTATACCGCTAAGGAACAACGAGCACGAGACCTAGCTGATGAACGCTCTAACGAGATTATCCGTAAGCTGACCCCTGAGCAACGTCGAGAAGCTCTCAACAACGGGACCCTTCTGTATCAGGATGACCCATA‑‑CGCTATGGAAGCACTCCGAGTCAAGACTGGTCGTAACGCTGCGTATCTTGTGGACGATGACGTTATGCAGCAGATAAAAGAGGGTGTCTTCCGTACTCGCGAAGAGATGGAAGAGTATCGCCATAGTCGCCTTCAAGAGGGCGCTAAGGTATACGc                                                                                                                                                                                                                         <  1:598660/301‑1 (MQ=255)`  
                                                                                   `cTGGTGCAGATATGTAGACCGCAAAGGAACAACGAGCACGAGACCTAGCTGATGAACGCTCTAACGAGATTATACGTAAGCTGACCCCTGAGCAACGTCGGGAAGCTCTCAACAACGGGACCCTTCTGTATCAGGAAGACCCATA‑‑CGCTATGGAAGCACTCCGAGTCAAGACTGGTCGTAACGCTGCGTATCTTGTGGACGATGACGTTATGCAGCAGATAAAAGAGGGTGTCTTCCGTACTCGCGAAGAGATGGAAGAGTATCGCCATAGTCGCCTTCAAGAGGGCGCTAAGGTATACGc                                                                                                                                                                                                                         <  1:347731/301‑1 (MQ=255)`  
                                                                                      `gTGCTGATATGTATACCGCTAAGGAACAACGAGCACGAGACCTAGCTGATGAACGCTCTAACGAGATTATCCGTAAGCTGACCCCTGAGCAACGTCGAGAAGCTCTCAACAACGGGACCCTTCTGTATCAGGATGACCCATA‑‑CGCTATGGAAGCACTCCGAGTCAAGACTGGTCGTAACGCTGCGTATCTTGTGGACGATGACGTTATGCAGCAGATAAAAGAGGGTGTCTTCCGTACTCGCGAAGAGATGGAAGAGTATCGCCATAGTCGCCTTCAAGAGGGCGCTAAGGTATACGCTgc                                                                                                                                                                                                                      >  1:438117/1‑300 (MQ=255)`  
                                                                                      `gTGCCGATATGTATACCGCTAAGGAACAACGAGCACGAGACCTAGCTGATGAACGCTCTAACGAGATTATCCGTAAGCTGACCCCTGAGCAACGTCGAGAAGCTCTCAACAACGGGACCCTTCTGTATCAGGATGACCCATA‑‑CGCTATGGAAGCACTCCGAGTCAAGACTGGTCGTAACGCTGCGTATCTTGTGGACGATGACGTTATGCAGCAGATAAAAGAGGGTGTCTTCCGTACTCGCGAAGAGATGGAAGAGTATCGCCATAGTCGCCTTCAAGAGGGCGGTAAGGTATACGCAGa                                                                                                                                                                                                                      >  1:190198/1‑301 (MQ=255)`  
                                                                                        `gCCGATATGTATACCGCTAAGGAACAACGAGCACGAGACCTAGCTGATGAACGCTCTAACGAGATTATCCGTAAGCTGACCCCTGAGCAACGTCGAGAAGCTCTCAACAACGGGACCCTTCTGTATCAGGATTACCCATA‑‑CGCTATGGAATCACTCCGAGTCAAGACTGGTCGTAACGCTGCGTATCTTGTGGACGATGACGTTATGCAGCAGATAAAAGAGGGTGTCTTCCGTACTCGCGAAGAGATGGAAGAGTATCGCCATAGTCGCCTTCAAGAGGGCGCTAAGGTATACGCTGAGc                                                                                                                                                                                                                    >  1:773581/1‑301 (MQ=255)`  
                                                                                        `gCCGATATGTATACCGCTAAGGAACAACGAGCACGAGACCTAGCTGATGAACGCTCTAACGAGATTATCCGTAAGCTGACCCCTGAGCAACGTCGAGAAGCTCTCAACAACGGGACCCTTCTGTATCAGGATGACCCATA‑‑CGCTATGGAAGCACTCCGAGTCAAGACTGGTCGTAACGCTGCGTATCTTGTGGACGATGACGTTATGCAGCAGATAAAAGAGGGTGTCTTCCGTACTCGCGAAGAGATGGAAGAGTATCGCCATAGTCGCCTTCAAGAGGGCGCTAAGGtctgtctcttat                                                                                                                                                                                                                    >  1:251458/1‑290 (MQ=255)`  
                                                                                             `tatGTATACCGCTAAGGAACAACGAGCACGAGACCTAGCTGATGAACGCTCTAACGAGATTATCCGTAAGCTGACCCCTGAGCAACGTCGAGAAGCTCTCAACAACGGGACCCTTCTGTATCAGGATGACCCATA‑‑CGCTATGGAAGCACTCCGAGTCAAGACTGGTCGTAACGCTGCGTATCTTGTGGACGATGACGTTATGCAGCAGATAAAAGAGGGTGTCTTCCGTACTCGCGAAGAGATGGAAGAGTATCGCCATAGTCGCCTTCAAGAGGGCGCTAAGGTATACGCTGAGCAGTTc                                                                                                                                                                                                               >  1:467171/1‑301 (MQ=255)`  
                                                                                             `tatGTATACCGCTAAGGAACAACGAGCACGAGACCTAGCTGATGAACGCTCTAACGAGATTATCCGTAAGCTGACCCCTGAGCAACGTCGAGAAGCTCTCAACAACGGGACCCTTCTGTATCAGGATGACCCATA‑‑CGCTATGGAAGCACTCCGAGTCAAGACTGGTCGTAACGCTGCGTATCTTGTGGACGATGACGTTATGCAGCAGATAAAAGAGGGTGTCTTCCGTACTCGCGAAGAGATGGAAGAGTATCGCCATAGTCGCCTTCAAGAGGGCGCTAAGGTATACGCTGAGCAGTTc                                                                                                                                                                                                               >  1:578217/1‑301 (MQ=255)`  
                                                                                             `tatGTATACCGCTAAGGAACAACGAGCACGAGACCTAGCTGATGAACGCTATAACGAGATTATCCGTAAGCTGACCCCTGAGCAACGTCGCGAAGCTCTCAACAACGGGACCCTTCTGTATCAGGATGACCCATA‑‑CGCTATGGAAGCACTCCGAGTCAAGACTGGTCGTAACGCTCCGTATCTTGTGGACGATGACGTTATGCAGCAGATAAACGAGGGTGTCTTCCGTACTCGCGAAGAGATGGAAGAGTATCGCCATAGTCGCCTTCAAGAGGGCGCTAAGGTATACGCTGAGcctgcc                                                                                                                                                                                                               >  1:804226/1‑296 (MQ=255)`  
                                                                                             `tatGTATACCGATAAGGAACAACGAGCACGAGACCTAGCTGATGAACGCTCTAACGAGATTATCCGTAAGCTGACCCCTGAGCAACGTCGAGAAGCTCTCAACAACGGGACCCTTCTGTATCAGGATGACCCATA‑‑CGCTATGGAAGCACTCCGAGTCAAGACTGGTCGTAACGCTGCGTATCTTGTGGACGATGACGTTATGCAGCAGATAAAAGAGGGTGTCTTCCGTACTCGCGAAGAGATGGAAGAGTATCGACATAGTCGCCTTCAAGAGGGCGCTAAggctgtctcttatacacat                                                                                                                                                                                                               >  1:225493/1‑284 (MQ=255)`  
                                                                                                `gTATACCGCTAAGGAACAACGAGCACGAGACCTAGCTGATGAACGCTCTAACGAGATTATCCGTAAGCTGACCCCTGAGCAACGTCGAGAAGCTCTCAACAACGGGACCCTTCTGTATCAGGATGACCCATA‑‑CGCTATGGAAGCACTCCGAGTCAAGACTGGTCGTAACGCTGCGTATCTTGTGGACGATGACGTTATGCAGCAGCTAAAAGAGGGTGTCTTCCGTACACGCGACGAGATGGAAGAGTATCGCCATAGTCGCCTTCAAGTGGGCGCTAAGGTATACGCTGCGCACTTCGGc                                                                                                                                                                                                            >  1:71075/1‑301 (MQ=255)`  
                                                                                                `gTATACCGCTAAGGAACAACGAGCACGAGACCTAGCTGATGAACGCTCTAACGAGATTATCCGTAAGCTGACCCCTGAGCAACGTCGAGAAGCTCTCAACAACGGGACCCTTCTGTATCAGGATGACCCATA‑‑CGCTATGGAAGCACTCCGAGTCAAGACTGGTCGTAACGCTGCGTATCTTGTGGACGATGACGTTATGCAGCAGATAAAAGAGGGTGTCTTCCGTACTTGCGAAGAGATGGTAGAGTATCGCCATCGTCGCCTTCAAGAGGGCGCTAAGGTATCCGCTTAGCAGTTCGGc                                                                                                                                                                                                            >  1:160742/1‑301 (MQ=255)`  
                                                                                                  `ataagagacaGGAACAACGAGCACGAGACCTAGCTGATGAACGCTCTAACGAGATTATCCGTAAGCTGACCCCTGAGCAACGTCGAGAAGCTCTCAACAACGGGACCCTTCTGTATCAGGATGACCCATA‑‑CGCTATGGAAGCACTCCGAGTCAAGACTGGTCGTAACGCTGCGTATCTTGTGGACGATGACGTTATGCAGCAGATAAAAGAGGGTGTCTTCCGTACTCGCGAAGAGATGGAAGAGTATCGCCATAGTCGCCTTCAAGAGGGCGCTAAGGTATACGCTGAGCAGTTCGGCAt                                                                                                                                                                                                          <  1:363030/292‑1 (MQ=255)`  
                                                                                                     `ccGCTAAGGAACAACGAGCACGAGACCTAGCTGATGAACGCTCTAACGAGATTATCCGTAAGCTGACCCCTGAGCAACGTCGAGAAGCTCTCAACAACGGGACCCTTCTGTATCAGGATGACCCATA‑‑CGCTATGGAAGCACTCCGAGTCAAGACTGGTCGTAACGCTGCGTATCTTGTGGACGATGACGTTATGCAGCAGATAAAAGATGGTGTCTTCCGTACTCGCGAAGAGATAGAAGAGTATCGCCATAGTCGCCTTCAAGAGGGCGCTAAGGTATACGCTGAGCAGTTCGGCATCGa                                                                                                                                                                                                       >  1:796416/1‑301 (MQ=255)`  
                                                                                                      `cGCTAAGGAACAACGAGCACGAGACCTAGCTGATGAACGCTCTAACGAGATTATCCGTAAGCTGACCCCTGAGCAACGTCGAGAAGCTCTCAACAACGGGACCCTTCTGTATCAGGATGACCCATA‑‑CGCTATGGAAGCACTCCGAGTCAAGACTGGTCGTAACGCTGCGTATCTTGTGGACGATGACGTTATGCAGCAGATAAAAGAGGGTGTCTTCCGTACTCGCGAAGAGATGGAAGAGTATCGCCATAGTCGCCTTCAAGAGGGCGCTAAGGTATACGCTGAGCAGTTCGGCATCGAc                                                                                                                                                                                                      <  1:541558/301‑1 (MQ=255)`  
                                                                                                      `acagaaGGAACAACGAGCACGAGACCTAGCTGATGAACGCTCTAACGAGATTATCCGTAAGCTGACCCCTGAGCAACGTCGAGAAGCTCTCAACAACGGGACCCTTCTGTATCAGGATGACCCATA‑‑CGCTATGGAAGCACTCCGAGTCAAGACTGGTCGTAACGCTGCGTATCTTGTGGACGATGACGTTATGCAGCAGATAAAAGAGGGTGTCTTCCGTACTCGCGAAGAGATGGAAGAGTATCGCCATAGTCGCCTTCAAGAGGGCGCTAAGGTATACGCTGAGCAGTTCGGCATCGAc                                                                                                                                                                                                      <  1:637597/297‑1 (MQ=255)`  
                                                                                                             `ggagatgtgtataagagacaggCTGATGAACGCTCTAACGAGATTATCCGTAAGCTGACCCCTGAGCAACGTCGAGAAGCTCTCAACAACGGGACCCTTCTGTATCAGGATGACCCATA‑‑CGCTATGGAAGCACTCCGAGTCAAGACTGGTCGTAACGCTGCGTATCTTGTGGACGATGACGTTATGCAGCAGATAAAAGAGGGTGTCTTCCGTACTCGCGAAGAGATGGAAGAGTATCGCCATAGTCGCCTTCAAGAGGGCGCTAAGGTATACGCTGAGCAGTTCGGCATCGACCCTGAgg                                                                                                                                                                                               <  1:812055/280‑1 (MQ=255)`  
                                                                                                             `aggagacagggaCGAGACCTAGCTGACGAACGCTCTAACGAGATTATCCGTAAGCTGACCCCTGAGTAACGTCGAGAAGCTCTGAACAACGGGACCCTTCTGTATCAGGATGACCCATA‑‑CGCTATGGAAGCAATCCGAGTCAAGACTGGTCGTAACGCTGCGTATCTTGTGGACGATGACGTTATGCAGCAGATAAAAGAGGGTGTCTGCCGTACTCGAGAAGAGATGGAAGAGTAGCGCCATAGTCGCCTTCAAGAGGGCGCTAAGGTATACGCTGAGCAGTTCGGCATCGACCCTGAgg                                                                                                                                                                                               <  1:52756/290‑1 (MQ=255)`  
                                                                                                                                        `gaACGCTCTAACGAGATTATCCGTAAGCTGACCCCTGAGCAACGTCGAGAAGCTCTCAACAACGGGACCCTTCTGTATCAGGATGACCCATA‑‑CGCTATGGAAGCACTCCGAGTCAAGACTGGTCGTAACGCTGCGTATCTTGTGGACGATGACGTTATGCAGCAGATAAAAGAGGGTGTCTTCCGTACTCGCGAAGAGATGGAAGAGTATCGCCATAGTCGCCTTCAAGAGGGCGCTAAGGTATACGCTGAGCAGTTCGGCATCGACCCTGAGGACGTTGATTATAAGCGTGGTTTCAACg                                                                                                                                                                    >  1:175047/1‑301 (MQ=255)`  
                                                                                                                                            `aagagacagagaTTATCCGTAAGCTGACCCCTGAGCAACGTCGAGAAGCTCTCAACCACGGGACCCTTCTGTATCAGGATGACCCATA‑‑CGCTATGGAAGCACTCCGAGTCAAGACTGGTCGTAACGCTGCGTATCTTGTGGACGATGACGTTATGCAGCAGATAAAAGAGGGTGTCTTCCGTACTCGCGAAGAGATGGAAGAGTATCGCCATAGTCGCCTTCAAGAGGGCGCTAAGGTATACGCTGAGCAGTTCGGCATCGACCCTGAGGACGTTGATTATCAGCGTGGTTTCAACGGGGa                                                                                                                                                                <  1:14680/293‑1 (MQ=255)`  
                                                                                                                                             `ctgtAATGAGATTATCCGTAAGCTGACCCCTGAGCAACGTCGAGAAGCTCTCAACAACGGGACCCTTCTGTATCAGGATGACCCATA‑‑CGCTATGGAAGCACTCCGAGTCAAGACTGGTCGTAACGCTGCGTATCTTGTGGACGATGACGTTATGCAGCAGATAAAAGAGGGTGTCTTCCGTACTCGCGAAGAGATGGAAGAGTATCGCCATAGTCGCCTTCAAGAGGGCGCTAAGGTATACGCTGAGCAGTTCGGCATCGACCCTGAGGACGTTGATTATCAGCGTGGTTTCAACGGGGAc                                                                                                                                                               >  1:749341/4‑301 (MQ=255)`  
                                                                                                                                             `ctctAACGAGATTATCCGTAAGCTGACCCCTGAGCAACGTCGAGAAGCTCTCAACAACGGGACCCTTCTGTATCAGGATGACCCATA‑‑CGCTATGGAAGCACTCCGAGTCAAGACTGGTCGTAACGCTGCGTATCTTGTGGACGATGACGTTATGCAGCAGATAAAAGAGGGTGTATTCCGTACTCGCGAAGAGATGGAAGAGTATCGCCATAGTCGCCTTCAAGAGGGCGCTAAGGTATACGCTGAGCAGTTCGGCATCGACCCTGAGGACGTTGATTATCAGCGTGGTTTCAACggggcc                                                                                                                                                               >  1:628396/1‑299 (MQ=255)`  
                                                                                                                                                     `agaTTATCCGTAAGCTGACCCCTGAGCAACGTCGAGAAGCTCTCAACAACGGGACCCTTCTGTATCAGGATGACCCATA‑‑CGCTATGGAAGCACTCCAAGTCAAGACTGGTCGTAACGCTGCGTATCTTGTGGACGATGACGTTATGCAGCAGATAAAAGAGGGTGTCTTCCTTACTCGCGAAGAGATGGAAGAGTATCGCCATAGTCGCCTTCAAGAGGGCGCTAAGGTATACGCTGAGCAGTTCGGCATCGACCCTGAGGACGTTGATTATcctgtctcttatacacatctcccagccca                                                                                                                                                       >  1:202552/1‑273 (MQ=255)`  
                                                                                                                                                      `aaagagacagaaGCTGACCCCTGGGCAACGTCGAGAAGCTCTCAACAACGGGACCCTTCTGTATCAGGATGACCCATA‑‑CGCTATGGAAGCACTCCGAGTCAAGACTGGTAGTAACGCTGCGTATCTTGTGGACGATGACGTTATGCAGCAGATAAAAGAGGGTGTCTTCCGTACTCGCGAAGAGATGGAAGAGTATCGCCATAGTCGCCTTCAAGAGGGCGCTAAGGTATACGCTGAGCAGTTCGGCATCGACCCTGAGGACGTTGATTATCAGCGTGGTTTCAACGGGGACATTACCGAg                                                                                                                                                      <  1:475498/291‑1 (MQ=255)`  
                                                                                                                                                        `ttATCCGTAAGCTGACCCCTGAGCAACGTCGAGAAGCTTCCAACAAAGGGACCTTTCTGTATCAGGATGACCCATC‑‑CGCTATGGAAGCACTCCGAGTCAAGACTGGTCGTAACGCTGCGTATCTTGTGGACGATGACGTTATGCAGCAGATAAAAGAGGGTGTCTTCCGTACTCGCGAAGAGATGGAAGAGTATCGACATAGTCGCCTTCAAGAGGGCGCTAAGGTATACGCTGAGCAGTTCGGCATCGACCCTGAGGACGTTGATTATCAGCGTGGTTTCAAAGGGTACATTACCGAGCg                                                                                                                                                    <  1:38388/301‑1 (MQ=255)`  
                                                                                                                                                           `tCCGTAAGCTGACCCCTGAGCAACGTCGAGAAGCTCTCAACAACGGGACCCTTCTGTATCAGGATGACCCATA‑‑CGCTATGGAAGCACTCCGAGTCAAGACTGGTCGTAACGCTGCGTATCTTGTGGACGATGACGTTATGCAGCAGATAACAGAGGGTGTCTTCCGTACTCGCGAAGAGATGGAAGAGTATCGCCATAGTCGCCTTCAAGAGGGCGCTAAGGTATACGCTGAGCAGTTCGGCATCGACCCTGAGGACGTTGATTATCAGCGTGGTTTCAACGGGGACATTACCGAGCGTaa                                                                                                                                                 >  1:823821/1‑301 (MQ=255)`  
                                                                                                                                                           `tCCGTAAGCTGACCCCTGAGCAACGTCGAGAAGCTCTCAACAACGGGACCCTTCTGTATCAGGATGACCCATA‑‑CGCTATGGAAGCACTCCGAGTCAAGACTGGTCGTAACGCTGCGTATCTTGTGGACGATGACGTTATGCAGCAGATAAAAGAGGGTGTCTTCCGTACTCGCGAAGAGATGGAAGAGTATCGCCATAGTCGCCTTCAAGAGGGCGCTAAGGTATACGCTGAGCAGTTCGGCATCGACCCTGAGGACGTTGATTATCAGCGGGGTTTCAACGGGGACATTACCGAGCGTaa                                                                                                                                                 >  1:218325/1‑301 (MQ=255)`  
                                                                                                                                                           `tCCGTAAGCTGACCCCTGAGCAACGTCGAGAAGCTCTCAACAACGGGACCCTTCTGTATCAGGAT‑ACCCATA‑‑CGCTATGGAAGCACTCCGAGTCAAGACTGGTCGTAACGCTGCGTATCTTGTGGACGATGACGTTATGCAGCAGATAAAAGAGGGTGTCTTCCGTACTCGCGAAGAGATGGAAGAGTATCGCCATAGTAGCCTTCAAGAGGGCGCTAAGGTATACGCTGAGCAGTTCGGCATCGACCCTGAGGACGGTGATTATCAGCGGGGTTTCAACGGGGACATTACCGAGCGTaaa                                                                                                                                                >  1:391459/1‑300 (MQ=255)`  
                                                                                                                                                            `ccGTAAGCTGACCCCTGAGCAACGTCGAGAAGCTCTCAACAACGGGACCCTTCGGTATCAGGATGACCCATA‑‑CGCTATGGAAGCACTCCGAGTCAAGACTGGTCGTAACGCTGCGTATCTTGTGGACGATGACGTTATGCAGCATATAAAAGAGGGTGTCTTCCGTACTCGCGAAGAGATGGAAGATTATCGCCATAGTCGCCTTCAAGAGGGCGCTAAGGTATACGCTGAGCAGTTCGGCATCGACCCTGAGGACGTTGATTATCAGCGTGGTTTCAACGGGGACATTACCGAGCGTAAc                                                                                                                                                <  1:518691/301‑1 (MQ=255)`  
                                                                                                                                                            `ccGGAAGCTGACCCCTGAGCAACGTCGAGAAGCTCTCAACAACGGGACCCTTCTGTATCAGGATGACCCATA‑‑CGCTATGGAAGCACTCCGAGTCAAGACTGGTCGTAACGCTGCGTATCTTGTGGACGATGACGTTATGCAGCAGATAAAAGAGGGTGTCTTCCGTACTCGCGAAGAGATGGAAGAGTATCGCCATAGTCGCCTTCAAGAGGGCGCTAAGGTATACGCTGAGCAGTTCGGCATCGACCCTGAGGACGTTGATTATCAGCGTGGTTTCAACGGGGACATTACCGAGCGTAAc                                                                                                                                                <  1:585356/301‑1 (MQ=255)`  
                                                                                                                                                             `cGTAAGCTGACCCCTGAGCAACGTCGAGAAGCTCTCAACAACGGGACCCTTCTGTATCAGGATGACCCATA‑‑CGCTATGGAAGCACTCCGAGTCAAGACTGGTCGTAACGCTGCGTATCTTGTGGACGATGACGTTATGCAGCAGATAAAAGAAGGTGTCTTCCGTACTCGCGAAGAGATGGAAGAGTATCGCCATAGTCGCCTTCAAGAGGGCGCTAAGGTATACGCTGGGCAGTTCGGCATCGACCCTGAGGACGTTGATTATCAGCGTGGTTTCAACGGGGACATTACCGAGCGTAACa                                                                                                                                               <  1:100097/301‑1 (MQ=255)`  
                                                                                                                                                                   `cTGACCCCTGAGCAACGTCGAGAAGCTCTCAACAACGGGACCCTTCTGTATCAGGATGACCCATA‑‑CGCTATGGAAGCACTCCGAGTCAAGACTGGTCGTAACGCTGCGTATCTTGTGGACGATGACGTTATGCAGCAGATAAAAGAGTGTGTCTTCCGTACTCGCGAAGAGATGGAAGAGTATCGCCATAGTCGCCTTCAAGAGGGCGCTAAGGTATACGCTGAGCAGTTCGGCATCGACCCTGAGGACGTTGATTATCAGCGTGGTTTCAACGGGGACATTACCGAGCGTAACATCTCGc                                                                                                                                         <  1:335749/301‑1 (MQ=255)`  
                                                                                                                                                                    `tGACCCCTGAGCAACGTCGAGAAGCTCTCAACAACGGGACCCTTCTGTATCAGGATGACCCATA‑‑CGCTATGGAAGCACTCCGAGTCAAGACTGGTCGTAACGCTGCGTATCTTGTGGACGATGACGTTATGCAGCAGATAAAAGAGGGTGTCTTCCGTACTCGCGAAGAGATGGAAGAGTATCGCCATAGTCGCCTTCAAGAGGGCGCTAAGGTATACGCTGAGCAGTTCGGCATCGACCCTGAGGACGTTGATTATCAGCGTGGTTTCAACGGGGACATTACCGAGCGTAACATCTCGCt                                                                                                                                        <  1:510305/301‑1 (MQ=255)`  
                                                                                                                                                                       `acccTGAGCAACGTCGAGAAGCTCTCAACAACGGGACCCTTCTGTATCAGGATGACCAATA‑‑CGCTATGGAAGCACTCCGAGTCAAGACTGGTAGTAACGCTGCGTATCTTGTGGACGATGACGTTATGCAGCAGATAAAAGAGGGTGTCTTCCGTACGCGCGAAGAGATGGAAGAGTATCGCCATAGGCGCCTTCAAGAGGGCGCTAAGGTATACGCTGAGCAGTTCGGCATCGACCCTGAGGAAGTTGATTAACAGCAGGGTTTCAACGGGGACATTAAGGAGCGTAACATCTGGCTGtt                                                                                                                                     >  1:452810/2‑300 (MQ=255)`  
                                                                                                                                                                             `aGCAACGTCGAGAAGCTCTCAACAACGGGACCCTTCTGTATCAGGATGACCCATA‑‑CGCTATGGAAGCACTCCGAGTCAAGACTGGTCGTAACGCTGCGTATCTTGTGGACGATGACGTTATGCAGCAGATAAAAGAGGGTGTCTTCCGTACTCGCGAAGAGATGGAAGAGTATCGCCATAGTCGCCTTCAAGAGGGCGCTAAGGTATACGCTGAGCAGTTCGGCATCGACCCTGAGGACGTTGATTATCAGCGTGGTTTCAACTGGGACATTACCGAGCGTAACATCTCGCTGTATGGTgc                                                                                                                               <  1:13133/301‑1 (MQ=255)`  
                                                                                                                                                                              `gCAACGTCGAGAAGCTCTCAACAACGGGACCCTTCTGTATCAGGATGACCCATA‑‑CGCTATGGAAGCACTCCGAGTCAAGACTGGTCGTAACGCTGCGTATCTTGTGGACGATGACGTTATGCAGCAGATAAAAGAGGGTGTCTTCCGTACTCGCGAAGAGATGGAAGAGTATCGCCATAGTCGCCTTCAAGAGGGCGCTAAGGTATACGCTGAGCAGTTCGGCATCGACCCTGAGGACGTTGATTATCAGCGTGGTTTCAACGGGGACATTACCGACCGTAACATCTCGCTGTAtctgtct                                                                                                                              >  1:489589/1‑295 (MQ=255)`  
                                                                                                                                                                                      `gagaAGCTCTCAACAACGGGACCCTTCTGTATCAGGATGACCCATA‑‑CGCTATGGAAGCACTCCGAGTCAAGACTGGTCGTAACGCTGCGTATCTTGTGGACGATGACGTTATGCAGCAGATAAAAGAGGGTGTCTTCCGTACTCTCGAAGAGATGGAAGAGTATCGCCATAGTCGCCTTCAAGAGGGCGCTAAGGTATACGCTGAGCAGTTCGGCATCGACCCTGAGGACGTTGATTATCAGCGTGGTTTCAACGGGGACATTACCGAGCGTAAcctgtctcttatacacatctccgagcc                                                                                                                      >  1:27012/1‑275 (MQ=255)`  
                                                                                                                                                                                       `agaAGCTCTCAACAACGGGACCCTTCGGTATCAGGATGACCCATA‑‑CGCTATGGAAGCACTCCGAGTCAAGACTGGTCGTAACGCTGCGTATCTTGTGGACGATGACGTTATGCAGCAGATAAAAGAGGGTGTCTTCCGTACTCGCGAAGAGATGGAAGAGTATCGCCATAGTCGCCTTCAAGAGGGCGCTAAGGTATACGCTGAGCAGTTCGGCATCGACCCTGAGGACGTTGATTATCAGCGTGGTTTCAACGGGGACATTACCGAGCGTAACATCTCGCTGTATGGTGCGCATGATAAc                                                                                                                     <  1:92242/301‑1 (MQ=255)`  
                                                                                                                                                                                                   `caacGGGACCCTTCTGTATCAGGATGACCCATA‑‑CGCTATGGAAGCACTCCGAGTCAAGTCTGGTCGTAACGCTGCGTATCTTGTGGACGATGACGTTATGCAGCAGATAAAAGAGGGTGTCTTCCGTACTCGCGAAGAGATGGAAGAGTATCGCCATAGTCGCCTTCAAGAGGGCGCTAAGGTATACGCTGAGCAGTTCGGCATCGACCCTGAGTACGTTGATTATCAGCGTGGTTTCAACGGGGACATTACCGAGCGTAACATCTCGCTGTATGGTGCGCATGATAACTTCTTGATCCAg                                                                                                         >  1:742646/1‑301 (MQ=255)`  
                                                                                                                                                                                                             `cTTCTGTATCAGGATGACCCATA‑‑CGCTATGGAAGCACTCCGAGTCAAGACTGGTCGTAACGCTGCGTATCTTGTGGACGATGACGTTATGCAGCAGATAAAAGAGGGTGTCTTCCGTACTCGCGAAGAGATGGAAGAGTATCGCCATAGTCGCCTTCAAGAGGGCGCTAAGGTATACGCTGAGCAGTTCGTCATCGACCCTGAGGACGTTGATTATCAGCGTGGTTTCAACGGGGACATTACCGAGCGTAACATCTCGCTGTATGGTGCGCATAATAACTTCTTGATCCAGCAAGCTCAGa                                                                                               >  1:670436/1‑301 (MQ=255)`  
                                                                                                                                                                                                              `ttCTGTATCAGGATGACCCATA‑‑CGCTATGGAAGCACTCCGAGTCAAGACTGGTCGTAACGCTGCGTATCTTGTGGACGATGACGTTATGCAGCAGATAAAAGAGGGTGTCTTCCGTACTCGCGAAGAGATGGAAGAGTATCGCCATAGTCGCCTTCAAGAGGGCGCTAAGGTATACGCTGAGCAGTTCGGCATCGACCCTGAGGACGTTGATTATCAGCGTGGTTTCAACGGGGACATTACCGAGCGTAACATCTCGCTGTCTGGTGCGCATGATAACTTCTTTAGCCAGCAAGCTcctgt                                                                                              >  1:422572/1‑297 (MQ=255)`  
                                                                                                                                                                                                               `tCTGTATCAGGATGACCCATA‑‑CGCTATGGAAGCACTCCGAGTCAAGACTGGTCGTAACGCTGCGTATCTTGTGGACGATGACGTTATGCAGCAGATAAAAGAGGGTGTCTTCCGTACTCGCGAAGAGATGGAAGAGTATCGCCATAGTCGCCTTCAAGAGGGCGCTAAGGTATACGCTGAGCAGTTCGGCATCGACCCTGAGGACGTTGATTATCAGCGTGGTTTCAACGGGGACATTACCGAGCGTAACATCTCGCTGTATGGTGCGCATGATAACTTCTTGAGCCAGCAAGCTCAGAAg                                                                                             <  1:789374/301‑1 (MQ=255)`  
                                                                                                                                                                                                               `tCTGTATCAGGATGACCCATA‑‑CGCTATGGAAGCACTCCGAGTCAAGACTGGTCGTAACGCTGCGTATCTTGTGGACGATGACGTTATGCAGCAGATAAAAGAGGGTGTCTTCCGTACTCGCGAAGAGATGGAAGAGTATCGCCATAGTCGCCTTCAAGAGGGCGCTAAGGTATACGATGAGCAGTTCGGCATCGACCCTGAGGACGTTGATTATCAGCGTGGTTTCAACGGGGACATTACCGAGCGTAACATCTCGCTGTATGGTGCGCATGATAACTTCTTGAGCCAGCAAGCTCAGAAg                                                                                             <  1:407471/301‑1 (MQ=255)`  
                                                                                                                                                                                                               `tCTGTATCAGGATGACCAATA‑‑CGCTATGGAAGAACTCCGAGTCAAGACTGGTCGTAACGCTGCGTATCTTGTGGACGATGACGTTATGCAGCAGATAAAAGAGGGTGTCTGCCGTACTCGCGAAGAGATGGAAGAGTATCGCCATAGTCGCCTTCAAGAGGGCGATAAGGTATACGCTGAGCAGTTCGGCATCGACCCTGAGGACGTTGATTATCAGCGTGGTTTCAACGGGGACATTACCGAGCGTAACATCTCGCTGTATGGTGCGCATGATAACTTCTTGAGCCAGCAAGCTCAGAAg                                                                                             <  1:116656/301‑1 (MQ=255)`  
                                                                                                                                                                                                                   `tATCAGTATGACCCATA‑‑CGCTATGGAAGCACTCCGAGTCAAGACTGGTCGTAACGCTGCGTATCTTGTGGACGATGACGTTATGCAGCAGATAAAAGAGGGTGTCTTCCGTACTCGCGAAGAGATGGAAGAGTATCGCCATAGTCGCCTTCAAGAGGGCGCTAAGGTATACGCTGAGCAGTTCGGCATCGACCCTGAGGACGTTGATTATCAGCGTGGTTTCAACGGGGACATTACCGAGCGTAACATCTCGCTGTATGGTGCGCATGATAACTTCTTGAGCCAGCAAGCTCAGAAGGgcg                                                                                         <  1:241701/301‑1 (MQ=255)`  
                                                                                                                                                                                                                        `ggATGACCCATA‑‑CGCTATGGAAGCACTCCGAGTCAAGACTGGTCGTAACGCTGCGTATCTTGTGGACGATGACGTTATGCAGCAGATAAAAGAGGGTGTCTTCCGTACTCGCGAAGAGATGGAAGAGTATCGCCATAGTCGCCTTCAAGAGGGCGCTAAGGTATACGCTGAGCAGTTCGGCATCGACCCTGAGGACGTTGATTATCAGCGTGGTTTCAACGGGGACATTACCGAGCGTAACATCTCGCTGTATGGTGCGCATGATAACTTCTTGAGCCAGCAAGCTCAGAAGGGCGCTATc                                                                                    >  1:399719/1‑301 (MQ=255)`  
                                                                                                                                                                                                                         `gATGACCCATA‑‑CGCTATGGAAGAACTCCGAGGCAAGACTGGTCGTAACGCTGCGTATCGTGTGGACGATGACGTTATGCAGCAGATAAAAGAGGGTGTCTTCCGTACTCGCGAAGAGATGGAAGAGTATCGCCATAGTCGCCTTCAAGAGGGCGCTAAGGTATACGCTGAGCAGTTCGGCATCGACCCTGAGGACGTTGATTATCAGCGTGGTTTCAACGGGGACATTACCGAGCGTAACATCTCGCTGTATGGTGCGCATGATAACTTCTTGAGCCAGCAAGCTCAGAAGGGCGCTAACa                                                                                   <  1:278269/301‑1 (MQ=255)`  
                                                                                                                                                                                                                              `cccATC‑‑CGCTATGGAAGCACTCCGAGTCAAGCCTGTTCGTAACGCTGCGTATCTTGTGGACGATGCCGTTATGCAGCAGATAAAAGAGGGTGTCTTCCGTACTCGCGAAGAGATGGAAGAGTATCGCCATAGTCGCCTTCAAGAGGGCGCTAAGGTATACGCTGAGCAGTTCGGCATCGACCCTGAGGACGTTGATTATCAGCGTGGTTTCAACGGGGACATTACCGAGCGTAACATCTCGCTGTATGGTGCGCATGATAACTTCTTGAGCCAGCAAGCTCAGAAGGGCGCTATCATGAAc                                                                              <  1:293509/301‑1 (MQ=255)`  
                                                                                                                                                                                                                              `cccATA‑‑CGCTATGGAAGCACTCCGAGTCAAGACTGGTCGTAACGCTGCGTATCTTGTGGACGATGACGTTATGCAGCAGATAAAAGAGGGTGTCTTCCGTACTCGCGAAGAGATGGAAGAGTATCGCCATAGTCTCCTTCAAGAGGGCGCTAAGGTATACGCTGAGCAGTTCGGCATCGACCCTGAGGACGTTGATTATCAGCGTGGTTTCAACGGGGACATTACCGAGCGTAACATCTCGCTGTATGGTGCGCATGATAACTTCTTGAGCCAGCAAGCTCAGAAGGGCGCTATCATGaaa                                                                              >  1:718548/1‑300 (MQ=255)`  
                                                                                                                                                                                                                              `cccATA‑‑CGCTATGGAAGCACACCGAGTCAAGACTGGTCGTAACGCTGCGTATCTTGTGGACGATGACGTTATGCAGCAGATAAAAGAGGGTGTCTTCCGTACTCGCGAAGAGATGGAAGAGTATCGCCATAGTCGCCTGCAAGAGGGCGCTAAGGTATACGCTGAGCAGTTCGGCATCGACCCTGAGGACGTTGATTATCAGCGTGGTTTCAACGGGGACATTACCGAGCGTAACATCTCGCTGTATGGTGCGCATGATAACTTCTTGAGCCAGCAAGCTCAGAAGGGCGCTATCATGAAc                                                                              <  1:590583/301‑1 (MQ=255)`  
                                                                                                                                                                                                                                `agagacagCTATGGAAGCACTCCGAGTCAAGACTGGTCGTAACGCTGCGTATCTTGTGGACGATGACGTTATGCAGCAGATAAAAGAGGGTGTCTTCCGTACTCGCGAAGAGATGGAAGAGTATCGCCATAGTCGCCTTCAAGAGGGCGCTAAGGTATACGCTGAGCAGTTCGGCATCGACCCTGAGGACGTTGATTATCAGCGTGGTTTCAACGGGTACATTACCGAGCGTAACATCTCGCTGTATGGTGCGCATGATAACTTCTTGAGCCAGCAAGCTCAGAAGGGCGCTATCATGAAc                                                                              <  1:81773/294‑1 (MQ=255)`  
                                                                                                                                                                                                                                 `aTA‑‑CGCTATGGAAGCACTCCGAGTCAAGACTGGTCGTAACGCTGCGTATCTTGTGGACGATGACGTTATGCAGCAGATAAAAGAGGGTGTCTTCCGTACTCGCGAAGAGATGGAAGAGTATCGCCATAGTCGCCTTCAAGAGGGCGCTAAGGTATACGCTGAGCAGTTCGGCATCGACCCTGAGGACGTTGATTATCAGCGTGGTTTCAACGGGGACATTACCGAGCGTAACATCTCGCTGTATGGTGCGCATGATAACTTCTTGAGCCAGCAAGCTCAGAAGGGCGCTATCATGAACAGc                                                                           <  1:85389/301‑1 (MQ=255)`  
                                                                                                                                                                                                                                  `tA‑‑CGCTATGGAAGCACTCCGAGTCAAGACTGGTCGTAACGCTGCGTATCTTGTGGACGATGACGTTATGCAGCAGATAAAAGAGGGTGTCTTCCGTACTCGCGAAGAGATGGAAGAGTATCGCCATAGTCGCCTTCAAGAGGGCGCTAAGGTATACGCTGAGCAGTTCGGCATCGACCCTGAGGACGTTGATTATCAGCGTGGTTTCAACGGGGACATTACCAAGCGTAACATCTCGCTGTATGGTGCGCATGATAACTTCTTGAGCCAGCAAGCTCAGAAGGGCGCTATCATGAACAGcc                                                                          >  1:459630/1‑301 (MQ=255)`  
                                                                                                                                                                                                                                   `ggtgtataaggaacagcTCCGAGTCAAGACTGGTCGTAACGCTGCGTATCTTGTGGACGATGACGTTATGCAGCAGATAAAAGAGGGTGTCTTCCGTACTCGCGAAGAGATGGAAGAGTATCGCCATAGTCGCCTTCAAGAGGGCGCTAAGGTATACGCTGAGCAGTTCGGCATCGACCCTGAGGACGTTGATTATCAGCGTGGTTTCAACGGGGACATTACCGAGCGTAACATCTCGCTGTATGGTGCGCATGATAACTTCTTGAGCCAGCAAGCTCAGAAGGGCGCTATCATGAACAGc                                                                           <  1:555338/285‑1 (MQ=255)`  
                                                                                                                                                                                                                                        `cTATGGAAGCACTCCGAGTCAAGACTGGTCGTAACGCTGCGTATCTTGTGGACGATGACGTTATGCAGCAGATAAAAGAGGGTGTCTTCCGTACTCGCGAAGAGATGGAAGAGTATCGCCATAGTCGCCTTCAAGAGGGCGCTAAGGTATACGCTGAGCAGTTCGGCATCGACCCTGAGGACGTTGATTATCAGCGTGGTTTCAACGGGGACATTACCGAGCGTAACATCTCGCTGTATGGTTCGCATGATAACTTCTTTAGCCAGCAAGCTCAGAAGGGCGCTATCCTGAACAGCCGAGt                                                                      >  1:482773/1‑301 (MQ=255)`  
                                                                                                                                                                                                                                                       `gAGGCAAGACAGGTCGTAACGCGGCGTATCTTGGGGACGATGACGTTATGCAGCAGATAAAAGAGGGTGTCTGCCGTACTCGCGAAGAGATGGAAGAGTATCGCCATAGTCGCCTTCAAGAGGGCGCTAAGGTATACGCTGAGCAGTTCGGCATCGACCCTGAGGACGTTGATTATCAGCGTGGTTTCAACGGGGACATTACCGAGCGTAACATCTCGCTGTATGGTGCGCATGATAACTTCTTGAGCCAGCAAGCTCAGAAGGGAGCTATCATGAACAGCCGAGTGGAACTCAACGgtgt                                                       <  1:107675/301‑1 (MQ=255)`  
                                                                                                                                                                                                                                                                   `gTCGTAACGCTGCGTATCTTGTGGACGATGACGTTATGAAGCAGATAAAAGAGGGTGTCTTCCGTACTCGCGAAGAGATGGAAGAGTATCGCCATAGTCGCCTTCAAGAGGGCGCTAAGGTATACGATGAGCAGTTCGGCATCGACCCTGAGGACGTTGATTATCAGCGTGGTTTCAACGGGGACATTACCGAGCGTAACATCTCGCTGTATGGTGCACATGATAACCTCTTGAGCCAGCAAGCTCAGAAGGGCGCTATCATGAACAGCCGAGTGGAACTCAACGGTGTCCTTCAAGAccc                                           <  1:123625/301‑1 (MQ=255)`  
                                                                                                                                                                                                                                                                   `ataagagacaggCGTATCTTGTGGACGATTACGTTATGCAGCAGATAAAAGAGGGTGTCTTCCGTACTCGCGAAGAGATGGAAGAGTATCGCCATAGTCGCCTTCAAGAGGGCGCTAAGGTATACGCTGAGCAGTTCGGCATCGACCCTGAGGACGTTGATTATCAGCGTGGTTTCAACGGGGACATTACCGAGCGTAACATCTCGCTGTATGGTGCGCATGATAACTTCTTGAGCCAGCAAGCTCAGAAGGGCGCTATCATGAACAGCCGAGTGGAACTCAACGGTGTCCTTCAAGAccc                                           <  1:535383/290‑1 (MQ=255)`  
                                                                                                                                                                                                                                                                    `tCGTAACGCTGCGTATCTTGTGGACGATGACGTTATGCAGCAGATAAAAGAGGGTGTCTTCCGTACTCGCGAAGAGATGGAAGAGTATCGCCATAGTCGCCTTCAAGAGGGCGCTAAGGTATACGCTGAGCAGTTCGGCATCGACCCTGAGGACGTTGATTATCAGCGTGGTTTCAACGGGGACATTACCGAGCGTAACATCTCGCTGTATGGTGCGCATGATAACTTCTTGAGCCAGCAAGCTCAGAAGGGCGCTATCATGAACAGCCGAGTGGAACTCAACGGTGGCCTTCAAGAcccg                                          >  1:700990/1‑300 (MQ=255)`  
                                                                                                                                                                                                                                                                              `gCGTATCTTGTGGACGATGACGTTATGCAGCAGATAAAAGAGGGTGTCTTCCGTACTCGCGAAGAGATGGAAGAGTATCGCCATAGTCGCCTTCAAGAGGGCGCTAAGGTATACGCTGAGCAGTTCGGCATCGACCCTGAGGACGTTGATTATCAGCGTGGTTTCAACGGTGACATTACCGAGCGTAACATCTCGCTGTATGGTGCGCATGATAACTTCTTGAGCCAGCAAGCTCAGAAGGGCGCTATCATGAACAGCCGAGTGGAACTCAACGGTGTCCTTCAAGACCCTGATAtgctgc                                >  1:726839/1‑301 (MQ=255)`  
                                                                                                                                                                                                                                                                                `atgtgtataagagacaaaCGTTATGCAGCAGATAAAAGAGTGTGTCTTCCGTACTCGCGAAGAGATGGAAGAGTATCGCCATAGTCGCCTTCAAGAGGGCGCTAAGGTATACGCTGAGCAGTTCGGCATCGACCCTGAGGACGTTGATTATCAGCGTGGTTTCAACGGGGACATTACCGAGCGTAACATCTCGCTGTATGGTGCGCATGATAACTTCTTGAGCCAGCAAGCTCAGAAGGGCGCTATCATGAACAGCCGAGTGGAACTCAACGGTGTCCTTCAAGACCCTGATATGCTGcgt                              <  1:613070/284‑1 (MQ=255)`  
                                                                                                                                                                                                                                                                                   `tCTTGTGGACGATGACGTTATGCAGCAGATAAAAGAGGGTGTCTTCCGTACTCGCGAAGAGATGGAAGAGTATCGCCATAGTCGCCTTCAAGAGGGAGCTAAGGAATACGCTGAGCAGTTCGGCATCGACCCTGAGGACGTTGATTATCAGCGTGGTTTCAACGGGGACATTACCGAGCGTAACATCTCGCTGTATGGTGCGCATGATAACTGCTTGAGCCAGCAAGCTCAGAAGGGCGCTATCATGAACAGCCGAGTGGAAGTGAACGGTGTCCTTAAAGACCCTGATATGCTGcgtcgt                           >  1:49577/1‑301 (MQ=255)`  
                                                                                                                                                                                                                                                                                    `cTTGTGGACGATGACGTTATGCAGCAGATAAAAGAGGGTGTCTTCCGTACTCGCGAAGAGATGGAAGAGTATCGCCATAGTCGCCTTCAAGAGGGCGCTAAGGTATACGCTGAGCAGTTCGGCATCGACCCTGAGGACGTTGATTATCAGCGTGGTTTCAACGGGGACATTACCGAGCGTAACATCTCGCTGTATGGTGCGCATGATAACTTCTTGAGCCAGCAAGCTCAGAAGGGCGCTATCATGAACAGCCGAGTGGAACTCAACGGTGTCCTTCAAGACCCTGATATGCTGCgtcgtc                          >  1:258843/1‑301 (MQ=255)`  
                                                                                                                                                                                                                                                                                         `ggACTATGACGTTATGCAGCAGATAAAAGAGGGTGTCTTCCGGACTCGCGAAGAGATGGAAGAGTATCGCCATAGTCGCCTTCAAGAGGGCGCTAAGGTATACGCTGAGCAGTTCGGCATCGACCCTGAGGACGTTGATTATCAGCGTGGTTTCAACGGGGACATTACCGAGCGTAACATCTCGCTGTATGGTGCGCATGATAACTTCTTGAGCCAGCAAGCTCAGAAGGGCGCTATCATGAACAGCCGAGTGGAACTCAACGGTGTCCTTCAAGACCCTGATATGCTGCGTCGTCCAGAc                     <  1:341483/301‑1 (MQ=255)`  
                                                                                                                                                                                                                                                                                          `gaagATGACGGCATGAAGAAGATAAAAGAGGGTGGATTCGGGATAAGAGAAGGGATGGAAGAGTATCGCGATAGTCGGCTTCAAGAAGGCGCTAAGGTATACGCTGAGCAGTGCGGCATGGAACATGAGGAGGTTGAGTATATGCGTGGTTTCAACGGGGACATTAACGAGCGTAACATCTCGCTGTATGGTGCGCATGATAACTTCTTGAGCAAGCAAGGTCAGAAGTGAGCTACCATGAACAGCCGAGTGGAACTCAACGGTGTCCTTCAAGACCCTGATATGCTGCGTCGTCCAGAct                    <  1:444376/298‑1 (MQ=255)`  
                                                                                                                                                                                                                                                                                              `aTGACGTTATGCAGCAGATAAAAGAGGGTGTCTTCCGTACTCGCGAAGAGATGGAAGAGTATCGCCATAGTCGCCTTCAAGAGGGCGCTAAGGTATACGCTGAGCAGTTCGGCATCGACCCTGAGGACGTTGATTATCAGCGTGGTTTCAACGGGGACATTACCGAGCGTAACATCTCGCTGTATGGTTCGCATGATAACTTCTTGAGCCAGCAAGCTCAGAAGGGCGCTATCATGAACAGCCGAGTGGAACTCAACGGTGTCCTTCAAGACCCTGATATGCTGCGTCGTCCAGaactgtc                >  1:431122/1‑295 (MQ=255)`  
                                                                                                                                                                                                                                                                                              `aTGACGTTATGCAGCAGATAAAAGAGGGTGTCTTCCGTACTCGCGAAGAGATGGAAGAGTATCGCCATAGTCGCCTTCAAGAGGGCGCTAAGGTATACGCTGAGCAGTTCGGCATCGACCCTGAGGACGTTGATTATCAGCGTGGTTTCAACGGGGACATTACCGAGCGTAACATCTCGCTGTATGGTGCGCATGATAACTTCTTGAGCCAGCAAGCTCAGAAGGGCGCTATCATGAACAGCCGAGTGGAACTCAACGGTGTCCTTCAAGACCCTGATATGCTGCGTCGTCCAGACTctgc                >  1:300307/1‑301 (MQ=255)`  
                                                                                                                                                                                                                                                                                                   `gTTTTGCAGCAGATAAAAGAGTGTGTCTTCCGTACTCGCGAAGAGATGGAAGAGTATCGCCATAGTCGCCTTCAATAGGGCTCTAAGGTATACGCTGAGCAGTTCGGCATCGACCCTGAGGACGTTGATTATCAGCGTGGTTTCAACGGGGACATTACCGAGCGTAACATCTCGCTGTATGGTGCGCATGATAACTTCTTGAGCCAGCAAGCTCAGAAGAGCGCTATCATGAACAGCCGAGGGGAACTCAACGGTGTCCTTCAAGACCCTGATATGCTGCGTCGTCCAGACTCTGCTGAct           <  1:414413/301‑1 (MQ=255)`  
                                                                                                                                                                                                                                                                                                   `gTTATGCAGCAGATAAAAGAGGGTGTCTTCCGTACTCGCGAAGAGATGGAAGAGTATCGCCATAGTCGCCTTCAAGAGGGCGCTAAGGTATATGCTGAGCAGTTCGGCATCGACCCTGAGGACGTTGATTATCAGCGTGGTTTCAACGGGGACATTACCGAGCGTAACATCTCGCTGTATGGTGCGCATGATAACTTCTTGAGCCAGCAAGCTCAGAAGGGCGCTATCATGAACAGCCGAGTGGAACTCAACGGTGTCCTTCAAGACCCTGATATGCTGCGTCGTCCAGACTCTGCTGAct           <  1:736134/301‑1 (MQ=255)`  
                                                                                                                                                                                                                                                                                                    `ttATGCAGCAGATAAAAGAGGGTGTCTTCCGTACTCGCGAAGAGATGGAAGAGTATCGCCATAGTCGCCTTCAAGAGGGCGCTAAGGTATACGCTGAGCAGTTCGGCATCGACCCTGAGGACGTTGATTATCAGCGTGGTTTCAACGGGGACATTACCGAGCGTAACATCTCGCTGTATGGTGCGCATGATAACTTCTTGAGCCAGCAAGCTCAGAAGGGCGCTATCATGAACAGCCGAGTGGAACTCAACGGTGTCCTTCAAGACCCTGATATGCTGCGTCGTCCAGACTCTGCTGActt          >  1:312431/1‑301 (MQ=255)`  
                                                                                                                                                                                                                                                                                                      `aTGCAGCAGATAAAAGAGGGTGTCTTCCGTACTCGCGAAGAGATGGAAGAGTATCGCCATAGTCGCCTTCAAGAGGGCGCTAAGGTATACGCTGAGCAGTTCGGCATCGACCCTGAGGACGTTGATTATCAGCGTGGTTTCAACGGGGACATTACCGAGCGTAACATCTCGCTGTATGGTGCGCATGATAACTTCTTGAGCCAGCAAGCTCAGAAGGGCGCTATCATGCACAGCCGAGTGGAACTCAACGGTGTCCTTCAAGACCCTGATATGCTGCGTCGTCCAGACTCTGCTGActtct        >  1:662852/1‑301 (MQ=255)`  
                                                                                                                                                                                                                                                                                                        `gcagcaGATAAAAGAGGGTGTCTTCCGTACTCGCGAAGAGATGGAAGAGTATCGCCATAGTCGCCTTCAAGAGGGCGCTAAGGTATACGCTGAGCAGTTCGGCATCGACCCTGAGGACGTTGATTATCAGCGTGGTTTCAACGGGGACATTACCGAGCGTAACATCTCGCTGTATGGTGCGCATGATAACTTCTTGAGCCAGCAAGCTCAGAAGGGCGCTATCATGAACAGCCGAGTGGAACTCAACGGTGTCCTTCAAGACCCGGATATGCTGCGTCGTCCAGACTCTGCTGACTTCttt      >  1:57020/1‑301 (MQ=255)`  

                                                                                                                                                                                                                                                                                                            `|`                                                                                                                                                                                                                                                                                                              
`AGGTATGGGCTATCGTGCAGCAACCACTCAGGCCGAACAGCCAAGGTCAAGCCTATTGGACACCATTGGTCGGTTCGCTAAGGCTGGTGCCGATATGTATACCGCTAAGGAACAACGAGCACGAGACCTAGCTGATGAACGCTCTAACGAGATTATCCGTAAGCTGACCCCTGAGCAACGTCGAGAAGCTCTCAACAACGGGACCCTTCTGTATCAGGATGACCCATA‑‑CGCTATGGAAGCACTCCGAGTCAAGACTGGTCGTAACGCTGCGTATCTTGTGGACGATGACGTTATGCAGAAGATAAAAGAGGGTGTCTTCCGTACTCGCGAAGAGATGGAAGAGTATCGCCATAGTCGCCTTCAAGAGGGCGCTAAGGTATACGCTGAGCAGTTCGGCATCGACCCTGAGGACGTTGATTATCAGCGTGGTTTCAACGGGGACATTACCGAGCGTAACATCTCGCTGTATGGTGCGCATGATAACTTCTTGAGCCAGCAAGCTCAGAAGGGCGCTATCATGAACAGCCGAGTGGAACTCAACGGTGTCCTTCAAGACCCTGATATGCTGCGTCGTCCAGACTCTGCTGACTTCTTTGAGA  >  NC_001604.1/28393‑28991`

  

Alignment Legend

Aligned base mismatch/match (shaded by quality score): `ATCG/ATCG` `< 3 ≤` `ATCG/ATCG` `< 11 ≤` `ATCG/ATCG` `< 23 ≤` `ATCG/ATCG` `< 35 ≤` `ATCG/ATCG` `< 38 ≤` `ATCG/ATCG`

Unaligned base: `atcg`    Masked matching base: `atcg`    Alignment gap: `‑`    Deleted base: `‑`
